# Skin permeation and penetration of mometasone furoate in the presence of emollients: An ex vivo evaluation of clinical application protocols

**DOI:** 10.1002/ski2.215

**Published:** 2023-01-20

**Authors:** Mubinah T. Beebeejaun, Marc B. Brown, Victoria Hutter, Laura Kravitz, William J. McAuley

**Affiliations:** ^1^ Centre for Research in Topical Drug Delivery and Toxicology University of Hertfordshire Hatfield UK; ^2^ MedPharm Ltd. Guildford UK

## Abstract

**Background:**

Topical corticosteroids (TCS) and emollients are developed independently by the pharmaceutical industry but are often used together in practice. There is potential for the TCS and emollient formulations to interact on the skin surface affecting TCS absorption into the skin. Clinical guidelines acknowledge this issue but lack an evidence base and differ in their recommendations. There is a current clinical need to establish whether the application protocol employed for TCS and emollient products can impact delivery of TCS to the skin.

**Objectives:**

To investigate whether the sequence of application of a TCS and emollient and the time between their application can affect TCS skin absorption.

**Methods:**

The delivery of mometasone furoate (MF) to ex vivo human skin was evaluated following the application of Elocon cream either 5 or 30 min, before and after three different emollients. Mechanistic explanation of the changes in drug absorption was provided by modelling the skin permeation data and Raman microscopy of mixed Elocon cream and emollient formulations.

**Results:**

A circa fivefold difference in MF absorption was observed depending on the emollient and application protocol. Applying Elocon cream at short intervals in relation to Hydromol intensive significantly increased MF absorption regardless of the application protocol. In contrast, applying Elocon cream after Diprobase cream or ointment significantly reduced MF absorption relative to Elocon cream alone or when Elocon cream was applied before these emollients. The changes in drug absorption observed were attributed to the presence of emollients altering Elocon cream formulation performance through different mechanisms, including introduction of penetration enhancing excipients and inducing drug crystallization in the mixed TCS emollient layer on the skin surface.

**Conclusions:**

Emollients can affect MF absorption in different ways depending on the emollient and sequence of administration. Using a 30 min gap between product applications may not be sufficient to mitigate emollient effects on TCS absorption.

## Graphical abstract

Up to a fivefold difference in mometasone furoate absorption was observed with both increases and decreases in absorption occurring depending on the emollient and application protocol. The changes in drug absorption were attributed to the presence of emollients altering Elocon cream formulation performance through different mechanisms, including introduction of penetration enhancing excipients and inducing drug crystallization in the mixed Elocon‐emollient layer on the skin surface. Using a 30 min interval between product applications may not be sufficient to mitigate emollient effects on topical corticosteroid absorption.

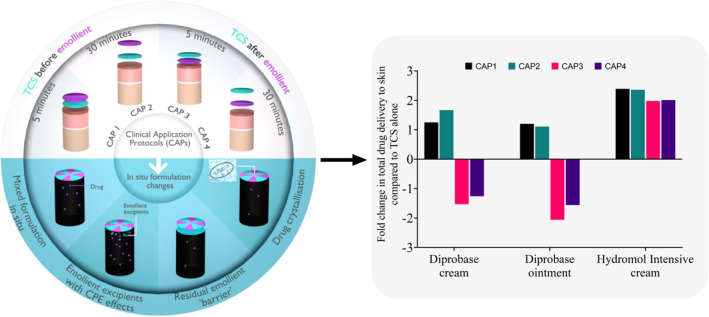

1

**What is already known about this topic?**

- When topical corticosteroids (TCS) and emollients are applied to the skin, the formulations may interact on the skin surface, potentially altering drug absorption. There is a lack of evidence to support clinical guidelines on how patients should use these products in relation to one another. Addressing this issue was identified as a research priority in 2013 by both atopic dermatitis patients and healthcare practitioners in the UK and remains unanswered.

**What does this study add?**

- Using an ex vivo human skin model, the presence of emollients was found to significantly alter the absorption of mometasone furoate (MF) from Elocon cream. The changes in absorption depended on both the emollient used and how the products were administered. Changes in drug absorption were the result different formulation effects including drug crystallization in the presence of emollient and the introduction of penetration enhancing excipients such as urea.

**What is the translational message?**

- Applying emollients at similar times as TCS can change drug absorption into the skin. It is likely that longer periods of time between product application for example, several hours is necessary to prevent any potential effect of the emollient on drug absorption. The data do not support the practice of applying emollients before a topical corticosteroid to aid its absorption.

## 2 INTRODUCTION

Topical corticosteroids (TCS) and emollients are commonly prescribed for the management of inflammatory skin conditions such as atopic dermatitis. These products are developed independently by the pharmaceutical industry, but clinical guidance often recommends the use of both products. When TCS and emollients are applied to the skin at similar times, the TCS formulation may effectively be altered on the skin surface, potentially altering drug absorption. Guidelines from professional bodies often recommend separating the application of TCS and emollients on the skin, however, these are derived from clinical judgement rather than evidence and there is considerable disparity in guidance for the use of these products together.^1^ They differ with respect to (i) whether the TCS or emollient should be applied first,^2^, ^3^ (ii) the time interval between application of the two products, which ranges from ‘as soon as absorbed’ to 60 min^4^, ^5^ and (iii) whether consideration should be made for the emollient formulation type (ointment or cream).^6^ These application recommendations can be complex, time consuming and without clear guidance, patients may apply products in quick succession to save time, with unknown consequences for treatment.^7^ In 2013, addressing the best way to use TCS and emollients was identified as a top research priority by both atopic dermatitis patients and healthcare practitioners in the UK; this remains unanswered.^8^, ^9^ A challenge to providing evidence on this topic is the large number of TCS and emollients that are used in practice. Clinical studies evaluating the effects of different application regimens for a wide range of products are prohibitively expensive. An alternative is ex vivo testing of drug absorption into the skin using Franz cells. This is widely adopted by the pharmaceutical industry to understand the impact of an array of formulation factors on drug delivery to the skin, the demonstration of bioequivalence between formulations and can offer insight into the interplay occurring between emollients and TCSs in situ.^10^ The approach has recently been used to provide data for post approval, medical affairs support on how to apply crisaborole ointment in relation to topical emollients.^11^ In this work, the impact of altering the order of, and time interval between, applications of Elocon cream with three emollients on mometasone furoate (MF) delivery to ex vivo human skin was evaluated, alongside mechanistic investigations of changes in TCS absorption.

## 3 MATERIALS AND METHODS

### 3.1 Materials

Micronized MF (Ph Eur) was provided by MedPharm Ltd. (Guildford, UK). Elocon cream (0.1% w/w MF), Diprobase cream, Diprobase ointment, and Hydromol Intensive cream were acquired from the University of Hertfordshire Campus Pharmacy (Hertfordshire, UK). Phosphate buffered saline (PBS) tablets, acetonitrile (high performance liquid chromatography [HPLC] grade), absolute ethanol (99+%), titanium dioxide, isopropyl myristate (IPM), urea and white soft paraffin were acquired from Fisher Scientific (Leicestershire, UK). Aluminium starch octenylsuccinate (DryFlo®) was provided by AzkoNobel (Warrington, UK). Non‐sterile, medical grade 0.002″ silicone membrane was purchased from Bioplexus (Los Angeles, USA).

### 3.2 Quantitative analysis of MF

Quantitative analysis of MF was achieved using HPLC with an Agilent 1260 Infinity system, a Hypersil™ C_18_ column (5 μm particle size, 250 × 4.6 mm; Phenomenex, UK) and a ultraviolet (UV) detection wavelength of 253 nm. The sample injection volume, flow rate and column temperature were 20 μl, 1 ml min^−1^ 21 ± 2°C, respectively. MF eluted at 19.6 min under the following mobile phase gradient conditions: 35% acetonitrile, 0–5 min; 35%–75% acetonitrile, 5–20 min; 75%–35% acetonitrile, 20–24 min, 35% acetonitrile, 24–26 min. The HPLC method was fit for purpose with respect to linearity (*r* ^2^ > 0.999), precision (<2% relative standard deviation [RSD]), accuracy (<2%) and sensitivity (the limit of detection was 0.09 μg ml^−1^ and the limit of quantification was 0.26 μg ml^−1^), in accordance with current International Council for Harmonisation of Technical Requirements for Pharmaceuticals for Human Use (ICH) guidelines.^12^

### 3.3 Skin preparation

Excised human scrotal skin was obtained with informed consent from gender reassignment surgeries following ethical approval from the South London Research Ethics Committee (ethics No. 10/H0807/51). Skin samples were removed from storage (−20°C) and left to thaw at ambient temperature, the subcutaneous fat was carefully removed using a scalpel and samples were stored at −20°C until required.

### 3.4 Ex vivo finite dose percutaneous absorption and skin distribution studies: Clinical application protocols

#### 3.4.1 Percutaneous absorption of MF

Calibrated Franz diffusion cells (Soham Scientific, UK) with an average volume of 3 ml and diameter of 1 cm were used. Skin samples were mounted in the Franz cells, the receiver chamber was filled with PBS and ethanol (7:3) and a magnetic flea was introduced for continuous stirring. Franz cells were equilibrated in a water bath at 37°C to achieve a skin surface temperature of 32°C then skin samples were dosed, using a calibrated positive displacement pipette, with 10 μl of Elocon cream alone or 10 μl of Elocon cream 5 min before an emollient (CAP1), 5 min after an emollient (CAP2), 30 min before an emollient (CAP3) or 30 min after an emollient (CAP4). The dose applied for each emollient (Diprobase cream, Diprobase ointment or Hydromol Intensive cream) was 10 μl. Alternatively, 20 μl of premixed TCS and emollient systems (1:1), or 10 μl of spiked Elocon cream formulations to which IPM or urea had been added, or 10 μl of a modified Elocon cream formulation, which had additional MF added to provide a concentration of 0.2% w/w MF were applied to the Franz cells. For testing under occluded conditions, the Franz cell donor chambers were occluded with parafilm immediately after dosing with 10 μl of Elocon cream.

#### 3.4.2 Skin distribution of MF

After 24 h, Franz cells were disassembled and the residual formulation was removed from the donor chamber and skin surface by three sequential wipes with cotton buds (a dry cotton bud, a cotton bud soaked in acetonitrile then a final dry cotton bud) and two tape strips (Scotch Tape strips, 3M Centre, USA) of the skin surface.

The epidermal and dermal layers of skin samples were heat separated at 60°C for 1 min,^13^ and the drug was extracted in acetonitrile before analysis.

#### 3.4.3 Raman microscopy of investigated formulations

Raman microscopy of particles observed in the formulations was performed using a Renishaw inVia Raman microscope (Renishaw, Gloucestershire, UK). Raman spectra were obtained using the ×100 long working distance magnification lens, a laser excitation wavelength of 785 nm, three accumulations per sample and an acquisition time of 10 s. Three replicate areas were scanned for each analysis and the single, most representative spectrum selected for presentation.

### 3.5 Data treatment and statistical analysis

Scientist^®^ 3.0 (Micromath Inc., Salt Lake City, UT, USA) was used to calculate the apparent partition (*Kh*) and diffusion (*D*/*h* ^2^) parameters when the Laplace transformation solution to Fick's second law, under finite dose conditions, was fit to the experimental permeation data sets.^14^, ^15^

Statistical analysis was performed using Prism 8.0 (GraphPad, USA). The Shapiro Wilk test was employed to determine the normality of all data sets. Non‐parametric analysis for multiple comparisons was performed using Kruskal–Wallis and a Mann–Whitney test applied for post hoc analysis. Statistical differences were accepted at the 95% confidence interval (*p* ≤ 0.05).

## 4 RESULTS

### 4.1 The impact of clinical application protocols on skin absorption of mometasone furoate

**FIGURE 1:**
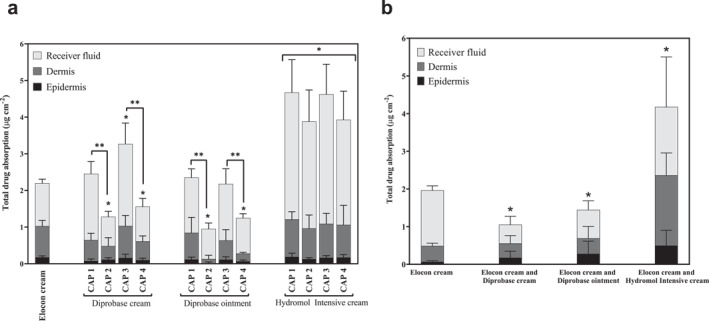
Skin distribution and percutaneous absorption of mometasone furoate. Drug delivery to the epidermis, dermis and receiver fluid from a finite dose of Elocon cream alone, Elocon cream applied according to a clinical application protocol (CAP) (a) and Elocon cream premixed with an emollient (b). CAP1: TCS 5 min before an emollient, CAP2: TCS 5 min after an emollient, CAP3: TCS 30 min before an emollient, CAP4: TCS 30 min after an emollient. The emollients were Diprobase cream, Diprobase ointment and Hydromol Intensive cream. Data are shown as mean ± SD (*n* = 6). * Denotes a significant difference (*p* < 0.05) when compared to the total drug recovered from Elocon cream alone. ** Denotes a significant difference (*p* < 0.05) when comparing the effect of the order of application on total drug delivery to the skin (CAP1 with CAP2, or CAP3 with CAP4).

Figure 1a shows MF delivery to the epidermis, dermis and the receiver fluid following the application of Elocon cream alone and with three emollients according to four clinical application protocols (CAPs). The CAPs were the application of the TCS before or after the emollients, with either a 5 or 30 min interval separating product applications. Figure 1b shows MF delivery from Elocon cream when mixed with each of the emollients immediately prior to application to the skin. When a TCS and topical emollient products are applied at similar times the products may interact to produce a new mixed formulation on the skin surface. Testing the drug delivery performance of Elocon cream diluted (1:1) with the emollients as premixed formulations enables an evaluation of whether delivery from the clinical application protocols is similar to that of the premixed (1:1) systems.

The total drug delivery (total drug content in the epidermis, dermis and receiver fluid) was used for statistical analysis as an indication of the change in total MF absorption compared to the application of Elocon cream alone.

Significant differences in total MF absorption were observed depending on the emollient and clinical application protocol used, with a circa fivefold difference between the highest and lowest values. Application of Hydromol Intensive cream increased MF delivery to the skin approximately twofold compared to Elocon cream alone, irrespective of the application protocol employed or whether the TCS and emollient where premixed prior to application to the skin (*p* < 0.05). In contrast, the application protocol impacted MF absorption when Diprobase cream or Diprobase ointment were the emollients. Applying Elocon cream 5 min after either Diprobase cream or Diprobase ointment reduced total drug delivery to the skin up to approximately 2.5‐fold, when compared to Elocon cream alone (*p* < 0.05). Increasing the time interval between product applications to 30 min was not sufficient to mitigate the emollient effects on significantly reducing drug delivery to the skin, with total drug delivery decreasing up to circa 1.5‐fold compared to Elocon cream alone (*p* < 0.05). The premixed Elocon cream and Diprobase cream or Diprobase ointment formulations reduced drug delivery to the skin (2‐fold and 1.5‐fold, respectively; *p* < 0.05) compared to Elocon cream alone, demonstrating behaviour consistent with the application of Elocon cream after these emollients. Applying Elocon cream before Diprobase cream or Diprobase ointment, however, largely resulted in unchanged total drug delivery to the skin when compared to the application of Elocon cream alone (*p* > 0.05); the exception to this was the application of Elocon cream 30 min before Diprobase cream which significantly increased MF absorption by 1.7‐fold compared to Elocon cream alone (*p* < 0.05).

### 4.2 Mechanistic evaluation of in situ formulation changes

**FIGURE 2:**
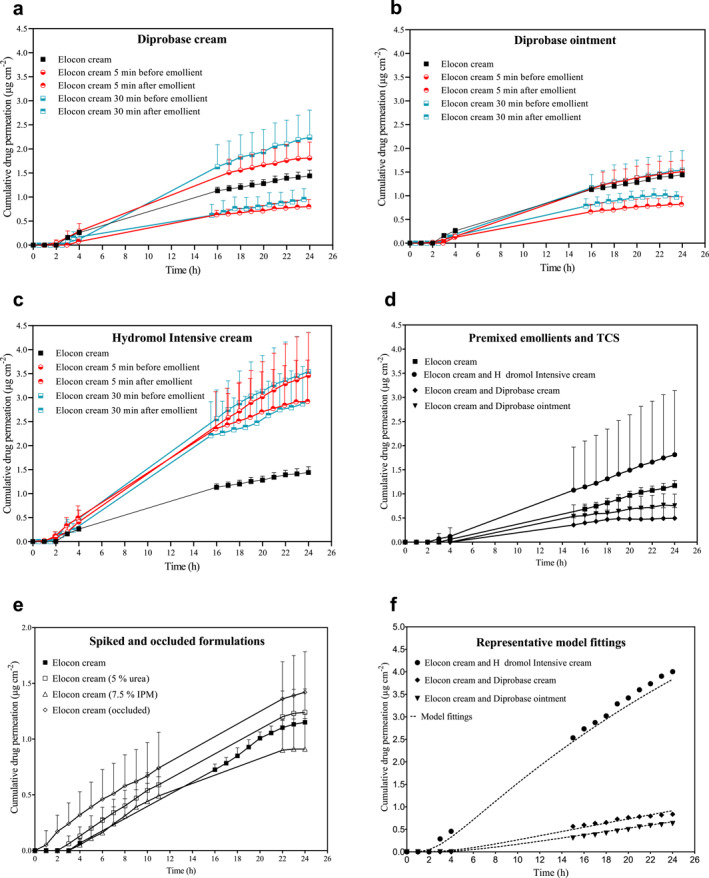
Mometasone furoate permeation across ex vivo human skin. The cumulative amount of mometasone furoate (μg cm^−2^) permeated over 24 h across human skin from Elocon cream when a finite dose was applied alone or: according to four clinical application protocols with (a) Diprobase cream; (b) Diprobase ointment; or (c) Hydromol Intensive cream; (d) premixed with Diprobase cream, Diprobase ointment or Hydromol Intensive cream (1:1); (e) under occlusion or from urea or IPM spiked formulations representative model fittings to the data are shown in (f). Data are shown as mean + SD (*n* = 6).

Plots of MF permeation across skin into the receiver fluid against time are presented in Figure 2 and show permeation profiles typical of finite dose experiments.^16^ These plots were modelled using Fick's second law to provide a mechanistic evaluation of emollient effects on drug delivery from Elocon cream. Representative model fittings are also shown in Figure 2 and show good fits to the data. The model enables changes in the rate of drug permeation across the skin, drug flux (*J*), to be separated into effects on drug partitioning (or transfer) from the formulation into the skin and drug diffusion through the skin to the receiver fluid, providing insight into how changes in drug absorption occur. The apparent partition co‐efficient (*Kh*), apparent diffusion co‐efficient (*D*/*h* ^2^) and pseudo steady state drug flux (*J*_ss_) obtained from modelling the permeation profiles of the different clinical application protocols are presented in Table 1.

**TABLE 1:** Skin permeation parameters for mometasone furoate when employing various clinical application protocols

Time interval	Product 1	Product 2	*D*/*h* ^2^ (cm)	*Kh* (h^−1^)	*J*_ss_ (μg cm^−2^ h^−1^)
	Elocon cream		3.29E‐02	3.87E‐03	1.29E‐05
			±2.95E‐03	±4.77E‐04	±2.90E‐06
5 min	Elocon cream	Diprobase cream	5.92E‐02^a^	2.13E‐03^a^	1.27E‐05
			±9.14E‐03	±1.11E‐04	±2.30E‐06
	Diprobase cream	Elocon cream	3.00E‐02	2.02E‐03^a^	6.07E‐06^a^
			±3.38E‐03	±8.88E‐05	±7.77E‐07
30 min	Elocon cream	Diprobase cream	3.87E‐02	4.81E‐03	1.87E‐05
			±5.98E‐03	±2.56E‐04	±3.61E‐06
	Diprobase cream	Elocon cream	3.13E‐02	2.39E‐03^a^	7.53E‐06^a^
			±4.20E‐03	±1.19E‐04	±1.29E‐06
5 min	Elocon cream	Diprobase ointment	4.60E‐02^a^	2.28E‐03	1.05E‐05
			±6.40E‐03	±1.27E‐04	±2.02E‐06
	Diprobase ointment	Elocon cream	3.10E‐02	2.01E‐03^a^	6.23E‐06^a^
			±4.24E‐03	±1.41E‐04	±1.09E‐06
30 min	Elocon cream	Diprobase ointment	4.66E‐02^a^	2.26E‐03^a^	1.0E‐05
			±9.21E‐03	±1.34E‐04	±2.51E‐06
	Diprobase ointment	Elocon cream	3.84E‐02	1.96E‐03^a^	7.51E‐06^a^
			±4.08E‐03	±7.61E‐05	±7.47E‐07
5 min	Elocon cream	Hydromol Intensive	4.16E‐02^a^	7.03E‐03^a^	2.97E‐05^a^
			±6.77E‐03	±4.23E‐04	±6.62E‐06
	Hydromol Intensive	Elocon cream	5.10E‐02^a^	4.80E‐03	2.44E‐05^a^
			±1.43E‐02	±3.61E‐04	±6.87E‐06
30 min	Elocon cream	Hydromol Intensive	4.22E‐02	7.74E‐03^a^	3.31E‐05^a^
			±9.01E‐03	±6.16E‐04	±9.43E‐06
	Hydromol Intensive	Elocon cream	2.94E‐02	9.32E‐03^a^	2.77E‐05^a^
			2.39E‐03	±9.62E‐04	±5.13E‐06

*Note*: Data are shown as mean ± SD (*n* = 6).

Abbreviations: *D*/*h* ^2^, estimated apparent diffusion co‐efficient; *J*_ss_, pseudo steady state drug flux; *Kh*, apparent partition co‐efficient.

^a^ Denotes a significant difference when *D*/*h* ^2^, *Kh* and *J*_ss_ values were compared to the respective permeation parameters for Elocon cream alone (*p* < 0.05).

The changes in *J*_ss_ largely reflect the change in total drug delivery to the skin, supporting the use of this analysis to interpret the mechanisms for changes in drug absorption. *J*_ss_ was significantly increased up to 2.6‐fold when Elocon cream was applied before or after Hydromol Intensive cream (*p* < 0.05). This was ascribed to changes in both *D*/*h* ^2^ and *Kh*, although effects on partitioning behaviour were usually larger. A similar effect was observed when Elocon cream was premixed with Hydromol Intensive cream (Table 2). In contrast, *J*_ss_ was reduced approximately twofold when Elocon cream was applied 5 or 30 min after Diprobase cream or Diprobase ointment (*p* < 0.05) which was attributed to reduced partitioning of MF into the skin (*p* < 0.05). A similar reduction in *J*_ss_ across skin was observed with the premixed formulations of Elocon cream and these two emollients (*p* < 0.05). When Elocon cream was applied before Diprobase cream and ointment, there was no significant change in *J*_ss_ compared to Elocon cream alone (*p* > 0.05). However, there were reductions in *Kh*, as seen when Elocon cream was applied after these emollients, but in this case the reductions in *Kh* were counteracted by increases in *D*/*h* ^2^ suggesting that more than one mechanism was affecting the drug permeation process.

**TABLE 2:** Skin permeation parameters for mometasone furoate when Elocon cream was applied in a premixed emollient system

	Applied dose of formulation (equivalent *C_v_*)	*D/h* ^2^ (cm)	*Kh* (h^−1^)	*J*_ss_ (μg cm^−2^ h^−1^)
Elocon cream	10 μl (0.1%)	2.59E‐02	3.51E‐03	9.08E‐06
		±2.08E‐03	±6.85E‐05	±6.32E‐07
Elocon cream and Diprobase cream	10 μl of TCS mixed with 10 μl of emollient (0.05%)	3.05E‐02	2.51E‐03^a^	3.88E‐06^a^
		±7.80E‐03	±1.48E‐04	±1.25E‐06
Elocon cream and Diprobase ointment		3.08E‐02	3.58E‐03	5.54E‐06^a^
		±7.30E‐03	±1.32E‐04	±1.43E‐06
Elocon cream and Hydromol Intensive cream		2.93E‐02	9.42E‐03^a^	1.46E‐05
		±1.41E‐02	±1.44E‐03	±9.41E‐06

*Note*: Data are shown as mean ± SD (*n* = 6).

Abbreviations: *C*_v_, drug concentration in vehicle; *D*/*h* ^2^, estimated apparent diffusion co‐efficient; *J*_ss_, pseudo steady state drug flux; *Kh*, apparent partition co‐efficient.

^a^ Denotes a significant difference when *D*/*h* ^2^, *Kh* and *J*_ss_ values were compared to the respective permeation parameters for Elocon cream alone (*p* < 0.05).

**FIGURE 3:**
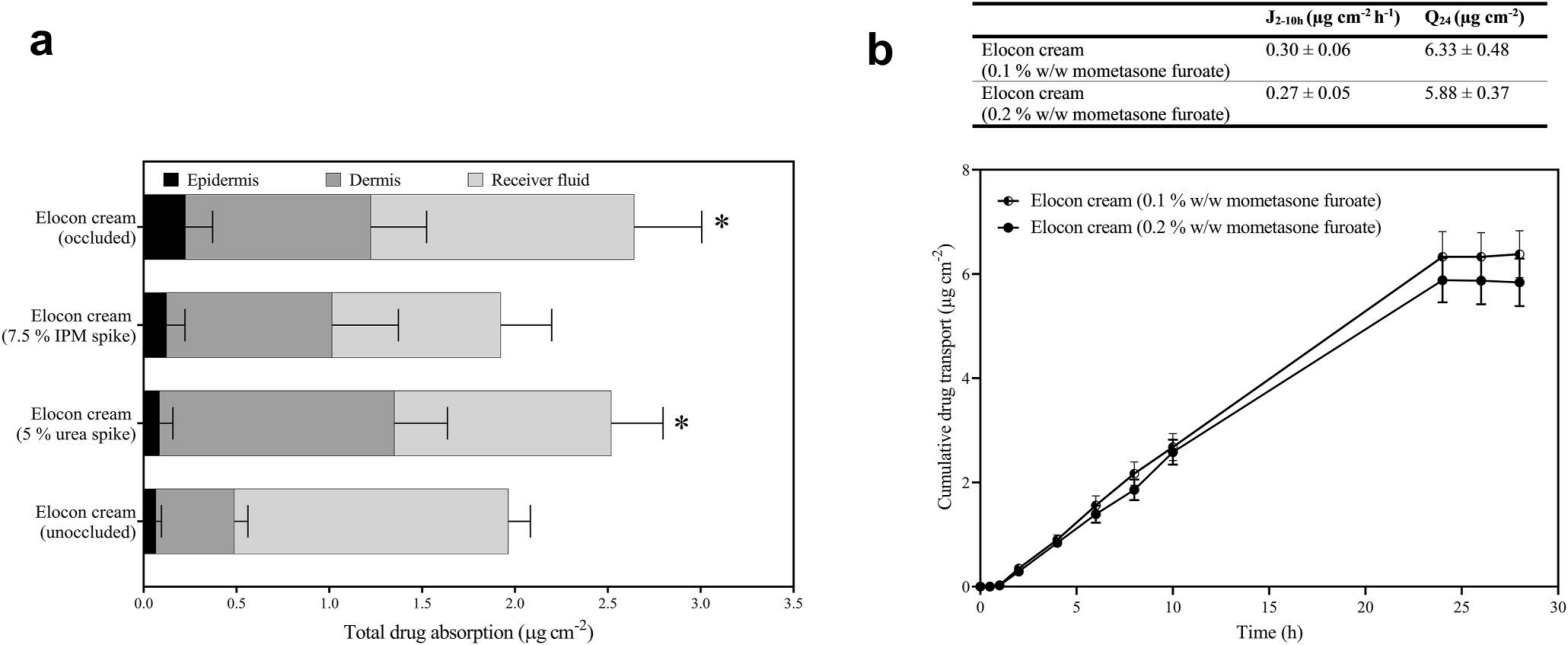
Isolation of emollient formulation effects on drug delivery to ex vivo skin and investigating the degree of drug saturation in Elocon cream. (a) Skin distribution and percutaneous absorption of mometasone furoate following the finite application of Elocon cream unoccluded, occluded and spiked with urea (5% w/w) or IPM (7.5% w/w). * Denotes a significant difference when the total drug delivery (epidermis, dermis and receiver fluid) was compared to Elocon cream (unoccluded) (*p* < 0.05). (b) Mometasone furoate (μg cm^−2^) transport across silicone membrane over 28 h from 0.1% w/w Elocon cream and 0.2% w/w Elocon cream following the application of an infinite dose of the formulations. * Denotes a significant difference when J2‐10 h or Q24 from 0.1% w/w Elocon cream was compared, respectively, to 0.2% w/w Elocon cream (*p* < 0.05). Data are shown as mean + SD (*n* = 6).

**FIGURE 4:**
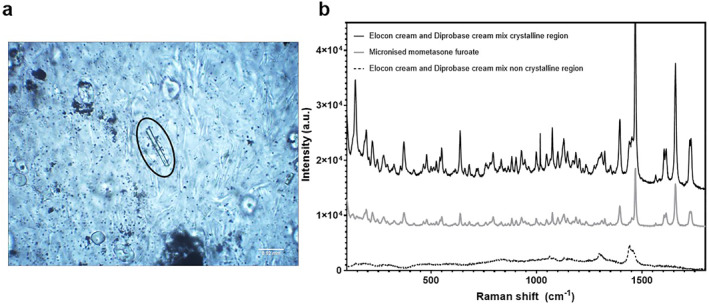
Mometasone furoate crystallization in the presence of Diprobase cream. (a) Light microscopy image (×20 magnification) of a drug crystal in a mixed Elocon cream and Diprobase cream formulation (1:1). (b) Raman spectra obtained from micronized mometasone furoate, crystalline and non‐crystalline regions of a mixed Elocon cream and Diprobase cream formulation (1:1).

To provide further insight into the mechanisms underlying the changes in MF absorption into the skin in the presence of emollients, further experiments were performed. Particular excipients contained within Hydromol Intensive cream, urea and isopropyl myristate, have demonstrated chemical penetration enhancing effects.^17^, ^18^ To investigate whether these excipients were increasing MF absorption from Elocon cream in the presence of Hydromol Intensive cream, Franz cell experiments were performed where Elocon cream was spiked with either urea or IPM (5% w/w or 7.5% w/w, respectively: Figure 3a, permeation profiles Figure 2e). Urea enhanced total drug delivery to the skin from Elocon cream approximately 1.3‐fold, when compared to the application of Elocon cream alone (*p* < 0.05), whereas IPM had no significant effect on total drug delivery (*p* > 0.05). Emollients have the potential to increase the occlusiveness of the MF vehicle on the skin surface. Figure 3a (permeation profile Figure 2e) also shows that occlusion of the Franz cell increased the delivery of MF to the skin from Elocon cream 1.3‐fold compared to non‐occluded conditions (*p* < 0.05). Changes in drug concentration of a formulation do not always proportionally impact the amount of drug delivered to the skin. Instead, an understanding of drug saturation in Elocon cream, is needed. Silicone membrane is particularly suitable to discriminate changes in drug saturation across different formulations and separate these effects from those of chemical penetration enhancers.^14^ This membrane was used to assess the saturation of MF in Elocon cream, through comparing drug transport from Elocon cream and a modified Elocon cream formulation to which additional MF had been added to increase its concentration from 0.1% to 0.2% w/w. No significant difference in drug flux between the marketed Elocon cream (0.1% w/w) and a 0.2% w/w MF formulation based on Elocon cream was observed (*p* > 0.05; Figure 3b). Mixed formulations containing Elocon cream and the different emollients were examined using light microscopy to understand whether the presence of emollient products induced physical changes in the TCS formulation behaviour. No changes were apparent when Elocon cream was mixed with Hydromol Intensive cream or Diprobase ointment. However crystalline particles that were not present in either Elocon cream or Diprobase cream were formed when these products were mixed (Figure 4a). Raman microscopy confirmed that these particles were MF crystals (Figure 4b).

## 5 DISCUSSION

This work provides for the first‐time mechanistic insight into changes in drug absorption that may occur when TCS are used in conjunction with emollients for the management of skin conditions. Elocon cream is a common, potent, TCS, Diprobase cream was within the top five prescribed emollients in the UK, Diprobase ointment, was selected to enable comparison between the effects of an emollient cream and ointment formulation and Hydromol Intensive is a frequently used urea containing emollient.^19^ The four application protocols were selected to reflect some of the current clinical application recommendations: applying the TCS before or after the emollients and with separation of the product applications by either a 5 or 30 min interval.

The data comparing MF transport across silicone membrane from Elocon cream and a modified 0.2% w/w Elocon cream indicated that drug saturation in Elocon cream was at or close its maximum value as increasing the drug concentration did not increase drug transport. Elocon cream also contains hexylene glycol, a solvent known to promote drug partitioning from the formulation.^20^, ^21^ Introduction of the emollients into Elocon cream either before application to the skin or on the skin surface may have altered drug or solvent saturation in the formulation and drug delivery to the skin in a variety of ways which are challenging to elucidate.

MF absorption from Elocon cream invariably increased with Hydromol Intensive cream regardless of the clinical application protocol or whether the products were mixed prior to applying them to the skin. IPM and urea, excipients in Hydromol Intensive cream, have been shown to enhance skin penetration of TCS.^17^, ^22^ Addition of 5% urea to Elocon cream increased MF absorption into the skin, suggesting that this excipient was partially responsible for the increased drug delivery observed with Hydromol Intensive cream. IPM may enhance drug delivery to the skin,^18^, ^23^ but did not improve MF absorption from Elocon cream here. However, IPM is also known to work synergistically with other chemical penetration enhancers^24^, ^25^ and therefore addition to Elocon cream on its own may not provide a full account of its impact on drug absorption when it is introduced through Hydromol Intensive.

Applying Elocon cream after Diprobase cream or Diprobase ointment reduced MF delivery to the skin through reducing partitioning of the drug from the formulation into the skin. Similar findings have been reported from a recent study where crisaborole ointment applied up to 15 min after a commercially available cream or ointment significantly reduced drug permeation and penetration to the skin.^11^ This behaviour was similar to that of the premixed Elocon cream and Diprobase cream and ointment systems, suggesting that formation of a physical emollient barrier layer by applying the emollient first does not explain the decreased MF delivery. Instead the reduced drug delivery to the skin is thought to be partially attributable to a reduction in MF saturation in the mixed formulation created in situ with Diprobase ointment.^26^ Changes in hexylene glycol saturation in the mixed vehicle compared to Elocon cream may also contribute to this effect. As a hydrophobic drug, reduction in MF saturation in a mixed vehicle is more likely to occur with the hydrophobic Diprobase ointment formulation which would be expected to display better solvent properties for the drug than the water containing Diprobase cream. In contrast, MF crystallization was observed to occur when Elocon cream was mixed with Diprobase cream, likely caused by the introduction of additional water from Diprobase cream, reducing its solubility in the mixed formulation. In this case the MF remained saturated in the mixed formulation system, however the decreased partitioning or transfer from the mixed formulation on the skin surface was a result of the crystallized drug no longer available for absorption, therefore reducing delivery to the skin.^18^, ^27^

Applying Elocon cream before Diprobase cream and Diprobase ointment did not cause the same reduction in drug delivery to the skin. Analysis of the permeation parameters indicated that MF diffusion through the skin was increased counteracting decreased drug partitioning from the formulation. Generally, occlusion of the skin surface is known to enhance drug permeation through increased hydration of the stratum corneum and emollient formulations have been shown to demonstrate occlusive properties.^28^ In this study, occluding the donor chamber of Franz cells increased MF absorption to the skin from Elocon cream. Thus, it is likely that for these emollients, the sequence of application impacts whether the occlusive effects counteract other negative effects on drug absorption to the skin.

In summary, this work provides evidence that applying topical products as per clinical guidance has the potential to significantly alter TCS formulation behaviour, expected drug delivery to the skin and may thus impact the clinically efficacy of the TCS. Allowing up to 30 min between product applications to minimize interaction between the products was not sufficient to mitigate emollient effects on TCS drug delivery to the skin. Contrary to guidance that the application of a TCS to well moisturized, thus hydrated, skin can increase the delivery of a TCS, the work presented herein established that applying a TCS after an emollient can significantly reduce TCS delivery to the skin. Indeed, it appears that longer time intervals between product applications, or prioritized application of the TCS may be necessary to avoid negative impacts on TCS absorption.

## CONFLICT OF INTEREST

The authors declared that they have no conflicts of interest to this work.

## AUTHOR CONTRIBUTIONS

**Mubinah T. Beebeejaun**: Conceptualization (Equal); Formal analysis (Equal); Investigation (Equal); Methodology (Equal); Visualization (Equal); Writing – original draft; Equal. **Marc B. Brown**: Conceptualization (Equal); Supervision (Equal); Writing – review & editing; Supporting. **Victoria Hutter**: Conceptualization (Equal); Supervision; Equal. **Laura Kravitz**: Conceptualization (Equal); Supervision; Equal. **William J. McAuley**: Conceptualization (Lead); Funding acquisition (Lead);Project administration (Equal); Supervision (Lead);Writing – review & editing (Lead).

## ETHICS STATEMENT

No human subjects were used in this study. Excised human was obtained with informed consent from surgeries following ethical approval from the South London Research Ethics Committee (ethics No. 10/H0807/51).

## Supporting information

Supporting Information S1

## Data Availability

Datasets related to this article can be found at https://www.research.herts.ac.uk/admin/editor/dk/atira/pure/api/shared/model/researchoutput/editor/contributiontojournaleditor.xhtml?scheme=%26type=%26id=27886507 hosted at the University of Hertfordshire Research Archives.
