# Supplementary material for: Skin permeation and penetration of mometasone furoate in the presence of emollients: An ex vivo evaluation of clinical application protocols
Source: Skin Health Dis. 2023 Jan 20;3(3):e215. doi: 10.1002/ski2.215 (PMC10233081; doi:10.1002/ski2.215)
Supplement: Supplementary file 1 — Supporting Information S1 [file SKI2-3-e215-s001.docx]

# Supplementary matERIALS

Supplementary methods section

## Mechanistic evaluation of investigated formulations

### Elocon cream applied under occlusion, Elocon cream spiked with IPM or urea, premixed TCS and emollient systems.

Premixed TCS and emollient systems (1:1) were prepared 1 h in advance of dosing by mixing equal quantities of Elocon cream with Diprobase cream, Diprobase ointment or Hydromol Intensive cream. Spiked Elocon cream formulations were prepared 1 h in advance of dosing by adding sufficient amounts of IPM or urea to Elocon cream to achieve a 7. 5 % w/w IPM or 5 % w/w urea spiked Elocon cream formulation. The formulations were mixed thoroughly until homogenous in appearance. Franz cell experiments were performed as per the ‘clinical application protocol’ studies. The skin samples were dosed with 10 μL of Elocon cream alone and the donor chamber occluded immediately with parafilm, 10 µl of the spiked formulations or 20 μL of the premixed systems using a positive displacement pipette.

### Investigating the degree of drug saturation in Elocon cream

A 0.2 % w/w Elocon cream formulation was prepared by adding MF into a vial with Elocon cream to achieve a final formulation strength of 0.2 % w/w MF. The formulation was placed in a water bath at 40 ºC and stirred overnight before equilibrating to room temperature. Franz cells (n=6) were assembled with silicone membrane, then the membrane was dosed with 1g of Elocon cream (0.1 % w/w) or modified Elocon cream (0.2 % w/w) by weight. The Franz cell study parameters were as detailed for the percutaneous absorption study.

## Data treatment and statistical analysis

Scientist^®^ 3.0 (Micromath Inc, Salt Lake City, UT, USA) was used to calculate the apparent partition (K*h)* and diffusion (D/*h*^2^) parameters when the Laplace transformation solution to Fick’s second law, under finite dose conditions, was fit to the experimental permeation data sets (Equation 1). The Laplace variable is denoted as ‘*s*’ in Equation 1. Estimations of K*h* (denoted as P_1_) and D/*h*^2^ (denoted as P_2_) were determined based on the following experimental parameters: Diffusional surface area (A), amount of drug applied (Q_0_), estimated membrane thickness (*h*) and the volume of formulation applied (V). The drug concentration in the formulation (set by Q_0_ and V) was set to 0.1 % for the application of Elocon cream alone and the clinical application protocols or 0.05 % when Elocon cream was applied in the premixed TCS and emollient systems.

$$\bar{Amount}=\frac{AP_{1}Q_{0}}{s\left[ V \sqrt{\frac{s}{P_{2}}}\sinh\sqrt{\frac{s}{P_{2}}}+P_{1}A\cosh\sqrt{\frac{s}{P_{2}}} \right]}$$

**Equation 1**

The pseudo steady state drug flux (J_ss_) for drug permeation were estimated as previously described by Oliveira et al. (2012b) using Equation 2.

$$J_{ss}=\frac{D}{\text{h}^{\text{2}}} x K\text{h} x C_{v}$$

**Equation 2**

The modelling was performed assuming that the drug concentration or thermodynamic activity in Elocon cream was unaffected when applied before or after an emollient, that is the drug concentration or thermodynamic activity was the same as that of the original product (0.1 % w/w). In contrast the modelling data presented in Table 2 for the premixed TCS and emollient formulations was performed assuming that the drug concentration or thermodynamic activity in Elocon cream was halved (0.05 % w/w). when applied in a premixed system as the original product had been diluted 1:1 with the emollient prior to application to the skin in the in this case. This approach enabled an evaluation of whether the clinical application protocols created a mixed system *in situ* that was altering the drug permeation parameters to the same extent as the respective premixed (1:1) systems.

Statistical analysis was performed using Prism 8.0 (GraphPad, USA). The Shapiro Wilk test was employed to determine the normality of all data sets. Non-parametric analysis for multiple comparisons was performed using Kruskal-Wallis and a Mann – Whitney test applied for post hoc analysis. Statistical differences were accepted at the 95 % confidence interval (*p* ≤ 0.05).

### Raman microscopy of investigated formulations

Raman microscopy of particles observed in the formulations was performed using a Renishaw inVia Raman microscope (Renishaw, Gloucestershire, UK), calibrated for peak position and intensity using a silicon reference block. Samples of Elocon cream alone and Elocon cream in a pre-mixed system with emollients (1:1) were mounted on Raman grade calcium fluoride slides for spectral analysis. To establish whether solid excipients within the formulations would interfere with crystalline structure analysis, spectra were also collected for titanium dioxide, aluminium starch octenylsuccinate (DryFlo^®^) and white soft paraffin. Raman spectra were obtained using the x 100 long working distance magnification lens, a laser excitation wavelength of 785 nm, three accumulations per sample and an acquisition time of 10 s. Three replicate areas were scanned for each analysis and the single, most representative spectrum selected for presentation.
